# Validation of a Novel Device to Measure and Provide Feedback on Sedentary Behavior

**DOI:** 10.1249/MSS.0000000000001458

**Published:** 2017-02-15

**Authors:** JASON M. R. GILL, NABEHA S. A. HAWARI, DOUGLAS J. MAXWELL, DAVID LOUDEN, NIKOS MOURSELAS, CHRISTOPHER BUNN, CINDY M. GRAY, HIDDE P. VAN DER PLOEG, KATE HUNT, ANNE MARTIN, SALLY WYKE, NANETTE MUTRIE

**Affiliations:** ^1^Institute of Cardiovascular and Medical Sciences, University of Glasgow, Glasgow, UNITED KINGDOM; ^2^PAL Technologies Ltd, Glasgow, UNITED KINGDOM; ^3^Institute of Health and Wellbeing, University of Glasgow, Glasgow, UNITED KINGDOM; ^4^Department of Public and Occupational Health, Amsterdam Public Health Research Institute, VU University Medical Center, Amsterdam, THE NETHERLANDS; ^5^MRC/CSO Social and Public Health Sciences Unit, Institute of Health and Wellbeing, University of Glasgow, Glasgow, UNITED KINGDOM; and ^6^Physical Activity for Health Research Centre, Institute for Sport, Physical Education and Health Sciences, University of Edinburgh, Edinburgh, UNITED KINGDOM

**Keywords:** SEDENTARY, SITTING, OBJECTIVE MEASUREMENT, VALIDATION, BEHAVIOR CHANGE

## Abstract

**Purpose:**

Pedometers, which enable self-monitoring of step counts, are effective in facilitating increases in physical activity. Similar devices which provide real-time feedback on sedentary (sitting) behavior are limited. This study aimed to develop and validate a novel device—the SitFIT—which could accurately measure and provide feedback on sedentary behavior and physical activity.

**Methods:**

The SitFIT is a triaxial accelerometer, developed by PAL Technologies, which is worn in the front trouser pocket. This enables tracking of thigh inclination and therefore differentiation between sitting and upright postures, as well as tracking of step count. It has a display to provide user feedback. To determine the validity of the SitFIT for measuring sedentary behavior and step counts, 21 men, age 30 to 65 yr, with body mass index 26.6 ± 3.9 kg·m^−2^ wore a SitFIT in a front trouser pocket and an activPAL accelerometer attached to their thigh for up to 7 d. Outputs from the SitFIT were compared with the activPAL, which was assumed to provide criterion standard measurements of sitting and step counts.

**Results:**

Mean step counts were approximately 4% lower with the SitFIT than activPAL, with correlation between the two methods being very high (*r* = 0.98) and no obvious bias from the line of equality (regression line, *y* = 1.0035*x* + 418.35). Mean sedentary time was approximately 5% higher with the SitFIT than activPAL, correlation between methods was high (*r* = 0.84), and the equation of the regression line was close to the line of equality (*y* = 0.8728*x* + 38.445).

**Conclusions:**

The SitFIT has excellent validity for measurement of free-living step counts and sedentary time and therefore addresses a clear need for a device that can be used as a tool to provide feedback on sedentary behavior to facilitate behavior change.

Sedentary behavior has been defined as waking activities in a sitting, reclining or lying posture with energy expenditure ≤1.5 METs (where 1 MET is resting energy expenditure) (1). Existing research, from both observational and experimental studies, demonstrate that high levels of sedentary behavior are associated with a range of adverse health outcomes including mortality, cardiovascular disease, type 2 diabetes and obesity (2–8), and that interventions which reduce sedentary behavior can induce positive changes to markers of health and disease risk (9–15). However, effective intervention tools to facilitate reductions in sedentary behavior are currently limited (16).

A considerable body of evidence from randomized controlled trials has shown that pedometer-based interventions—which enable individuals to self-monitor their physical activity level (i.e., steps taken per day), set physical activity targets and provide real-time feedback of progress toward their goal—are effective for increasing physical activity, and improving health outcomes in a range of population groups (17–19). Pedometers are also highly valued for self-monitoring by those taking part in behavioral interventions (20). There are a plethora of devices available which build on the pedometer to provide feedback of a number of indices of physical activity measurement, such as steps, distance travelled, and energy expenditure (21). However, consumer devices to enable the self-monitoring of free-living sedentary behavior are more limited, with the majority of devices using an acceleration-based, rather than posture-based, approach to estimate time spent sedentary (21,22). Thus, most currently available devices cannot distinguish between sitting and quiet standing, so cannot be used as a self-monitoring tool in interventions aiming to reduce time spent sitting. A small number of devices are available that use pressure sensors in a sock or shoe to determine standing or a pressure sensor on a chair to determine sitting (on a particular chair) (21), and one device worn on the lower back using an elasticated belt (originally developed to monitor posture) has also been used to monitor time spent sitting (21,22). Thus, devices available to monitor and provide feedback on time spent sitting under free-living conditions throughout the day are limited, and there is a clear need to develop and validate a device for the self-monitoring of sitting behavior, preferably in combination with step counts to target both physical activity and sedentary behavior with a single device.

The European Fans in Training (EuroFIT) study is a large-scale randomised controlled trial aiming to increase physical activity and reduce sedentary behavior over 12 months in middle-age male fans of football (soccer) clubs in England, The Netherlands, Norway and Portugal (23). To facilitate self-monitoring of physical activity and sedentary behavior in the EuroFIT trial (and future studies), we aimed to develop and validate a novel low-cost pocket-worn device with an integrated display—called the SitFIT—which could measure daily sedentary behavior and physical activity accurately, and provide real-time feedback to enable prompts for and self-monitoring of behavior change for both. This article describes the development of the SitFIT and the determination of its criterion validity (compared with the ActivPAL) for measurement of steps and sedentary time in a sample of adult males.

## METHODS

### 

#### Development of the SitFIT

**FIGURE 1:**
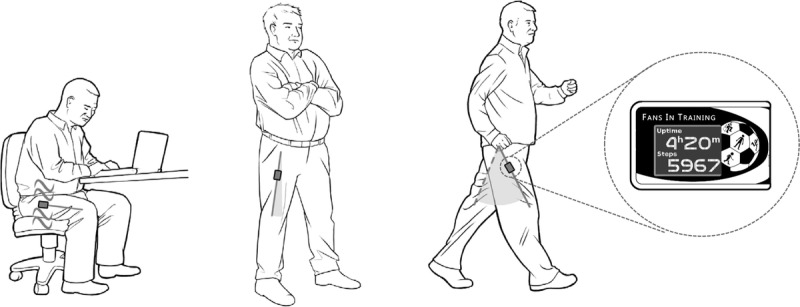
The pocket-worn SitFIT device during sitting, standing and stepping activities. The SitFIT tracks the orientation of the upper thigh, so changes orientation when posture changes from sitting to upright. The display provides real-time feedback of sitting (or upright) time and of step count.

The SitFIT is a triaxial accelerometer developed by PAL Technologies, which uses static and dynamic accelerations in the three orthogonal axes to calculate wear (and non-wear) time, posture allocation (upright or sedentary), transportation, and stepping. It has been designed to be worn in the front trouser pocket to enable the device orientation to track the inclination of the thigh allowing detection of sitting/lying and upright postures by assessment of the axes through which gravitational acceleration is detected (Fig. 1). This is the same concept underpinning the activPAL activity monitor (PAL Technologies, Glasgow, UK), a small triaxial accelerometer affixed to the front of the thigh, which is regarded as a criterion standard device for the measurement of free-living sitting behavior (in addition to its measurement of physical activity) because its thigh-based position is optimal for distinguishing between sitting and upright postures (24,25). However, because the activPAL is affixed to the thigh under clothing, it is not readily accessible; this, together with its lack of a display to provide feedback, makes it unsuitable for providing real-time feedback on sedentary behavior during everyday activities. The front trouser pocket location of the SitFIT tracks thigh inclination but provides the advantage of providing easy access for the user to enable provision of feedback. The pocket is also more likely to be acceptable for daily long-term wear than attachment to the thigh via a surgical dressing. Unlike the activPAL which has no facility to provide feedback on a screen on the device, the SitFIT was designed with a display to provide real-time visual feedback of stepping and sedentary/upright behaviors, a vibrotactile actuator to provide customisable haptic feedback of time spent sitting, and a Bluetooth SMART module to enable communication with external devices, such as smartphones, tablets, and PCs. The key characteristics of the ActivPAL and SitFIT are shown in Table 1.

**TABLE 1:**
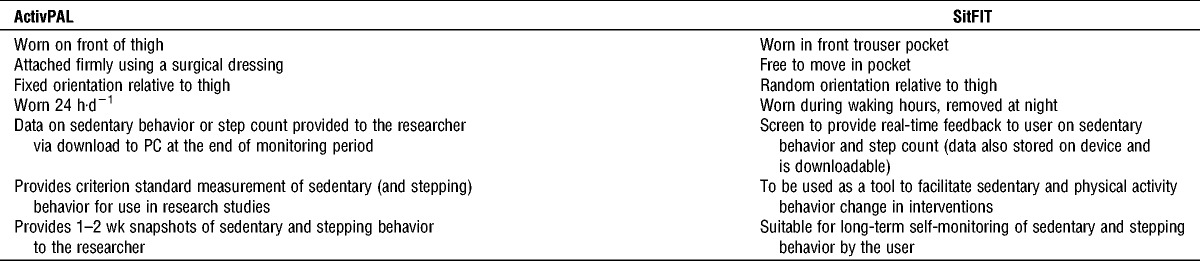
Characteristics of the ActivPAL and SitFIT.

Also, unlike the activPAL, which is held in a fixed orientation on the thigh, the SitFIT can move in the trouser pocket, thus changing its orientation relative to the thigh. To overcome this, algorithms were developed by PAL Technologies to allow the device to be carried at random orientations in the pocket and to rotate during use. The SitFIT produces outcomes that are mainly based on the device’s ability to count steps and to determine the wearer’s posture from its trouser pocket location. The SitFIT counts steps using all three (*X,Y,Z*) axes of space accelerations, with the step counting algorithm samplings each of the three axes separately 10 times every second. The algorithm looks for a swing leg phase expressed as a relative smooth variation of the axis acceleration value, followed by a sharp acceleration change attributed to heel strike. Depending on device orientation in the pocket, any axis can be dominant, hence the step count algorithm looks for all combinations of swing-heel strike patterns over three axes and their inversions. The count of steps is the sum of the steps counted across all axes, meaning that steps from all three axes are added but the same step is not counted more than once. A time-based filter is applied to cut off high frequency noise in the step counting arising from the device’s free movement inside the pocket that would otherwise produce extra step counts; practically, a refractory period is created between steps, preventing erroneous reporting of high frequency stepping. An automatic gain control feature is implemented based on interstep intervals that make the algorithm more sensitive during slow stepping. Additionally, there is a maximum period between two successive heel strikes that can lead to the registration of a step. Beyond this maximum, period step signals are regarded as individual noise bursts and do not contribute to step counting.

**FIGURE 2:**
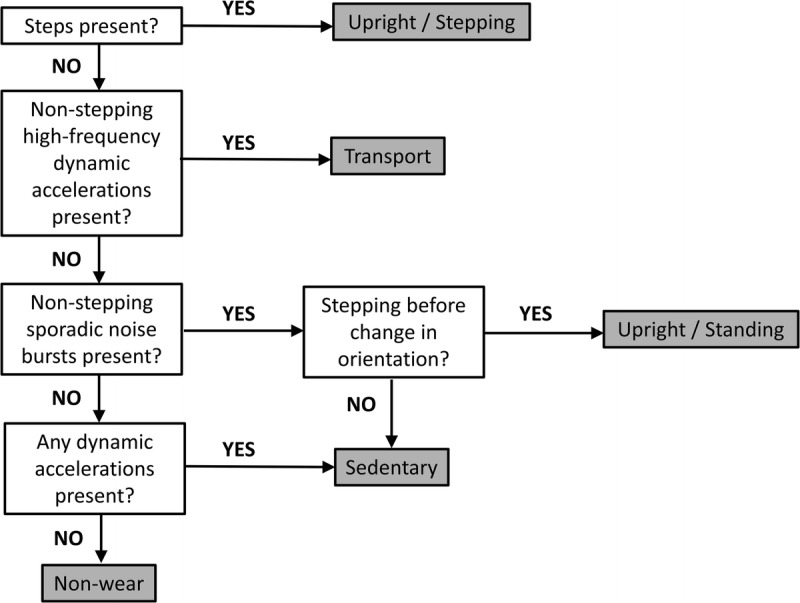
Flow diagram illustrating the algorithm for decision-rules used by the SitFIT to determine posture allocation.

The determination of posture from a randomly placed device in the pocket is a greater challenge than step counting. The posture estimation algorithm uses containers (i.e., periods where activity is of a single class) of upright, sedentary, transport and non-wear using historical and future criteria to set the limits for the sequential containers. The criteria used to characterise a container are: (a) the presence of steps, (b) high-frequency low-level background noise, (c) sporadic noise bursts, (d) a combination of changes to the static accelerations of the three axes. The highest weighted criterion to identify the upright container is the existence of steps. The algorithm identifies a container as upright when there are steps within it, and tracks back in time until the last sufficient change in static accelerations is found to indicate the change in posture. A prolonged period without significant dynamic accelerations is weighted towards a sedentary container. Any significant dynamic acceleration or stepping resets the weighting. A prolonged period totally without dynamic accelerations, after an identified sedentary period, weighs towards a non-wear container. Persistent, high-frequency, low-level dynamic accelerations without stepping are weighted toward a transport container. Sporadic noise bursts that do not constitute stepping are weighted towards upright (quiet standing). If no stepping is identified before a significant static acceleration change, the container is reassigned as sedentary. This algorithm is summarized in Figure 2.

#### Validation of stepping and sitting/upright time algorithms in free-living conditions

Once algorithms for detection of sitting versus upright time and step counts with the SitFIT were fully developed, we sought to validate their accuracy under real-world free-living conditions by comparing sitting time and step count outputs from the SitFIT with those from the activPAL, which was assumed to provide criterion standard measures of sitting time and step counts, over several days. To do this, we asked 21 men, age 30 to 65 yr, with body mass index of 26.6 ± 3.9 kg·m^−2^ who were willing to wear trousers with front pockets and had no contraindications to engaging in physical activity (as assessed by the Physical Activity Readiness Questionnaire) to each concurrently wear a SitFIT device in a front trouser pocket and an activPAL accelerometer attached to their thigh for up to 7 d. This participant group was chosen as the first intended use of the SitFIT was in the EuroFIT study which was a randomized controlled trial designed to increase physical activity and reduce sedentary behavior in overweight and obese middle-age male soccer fans (23). Participants were recruited via email invitation or word-of-mouth and were primarily employees of the University of Edinburgh. All provided written informed consent, and the study was approved by the Research Ethics Committee of the Moray House School of Education, University of Edinburgh.

Participants were instructed to affix an activPAL activity monitor (model activPAL3; PAL Technologies, Glasgow, UK) to the front of their thigh using a surgical dressing for 24 h·d^−1^ for 7 d. Over the same period, they were asked to carry a SitFIT device in their front trouser pocket during all waking hours, putting the device on as soon as they woke in the morning and removing it before they went to bed at night. Valid data were obtained for 7 d in 18 participants, 8 d in one participant, 6 d in one participant, and 5 d in one participant, providing a total of 145 valid days where SitFIT and activPAL data could be compared.

Data were processed using proprietary software developed by PAL Technologies, which summarized data in 5-min epochs throughout the day, quantifying the duration of time spent sitting (or lying), standing, stepping, and of non-wear, as well as the number of steps taken, in each epoch for both the activPAL and SitFIT devices. The software automatically detected periods of non-wear, using the algorithms described above, and data were cleaned to remove periods identified as non-wear for either device. Thus, data analysis only included the waking periods where both devices were worn: this step was necessary to ensure comparability of SitFIT and activPAL data, because SitFIT devices were removed at night. To determine whether it was necessary to account for nesting of multiple observation days per participant in our analysis, we explored the effect of including a term for “participant” in analysis of the linear regression between SitFIT and ActivPAL outputs for step count and sedentary time, and when comparing the mean difference in outputs between the two devices. This had no material effect on the findings (e.g., *r*^2^ for the correlation between SitFIT and ActivPAL sedentary time measurements was 0.7007 when all data points were considered independent and 0.7010 taking nesting into account. For step count, *r*^2^ was 0.9608 when all data points were considered independent and 0.9610 accounting for nesting). We therefore took the parsimonious approach of considering the each of the 145 observation days as independent data points in our data analysis. Cumulative sitting time and cumulative step count throughout each day was calculated for the SitFIT and activPAL devices for each of the 145 d, and mean ± SD values reported graphically. Mean (± SD) values for the difference in cumulative sitting time and step count were also shown in graphical form. Mean absolute errors for cumulative sitting time and step count were calculated as the mean of the absolute differences between SitFIT and ActivPAL measurements (i.e., ignoring the direction of error for each individual measurement). A Bland and Altman limits of agreement approach was used to ascertain bias and variability in the SitFIT measures of sitting time and step counts compared with the activPAL (26). The relationships between daily sitting time and step count outputs between activPAL and SitFIT were assessed by plotting scatter graphs and assessing Pearson correlations (*r*) between the two measures and proximity of the relationship to the line of equality (*y* = *x*).

## RESULTS

**FIGURE 3:**
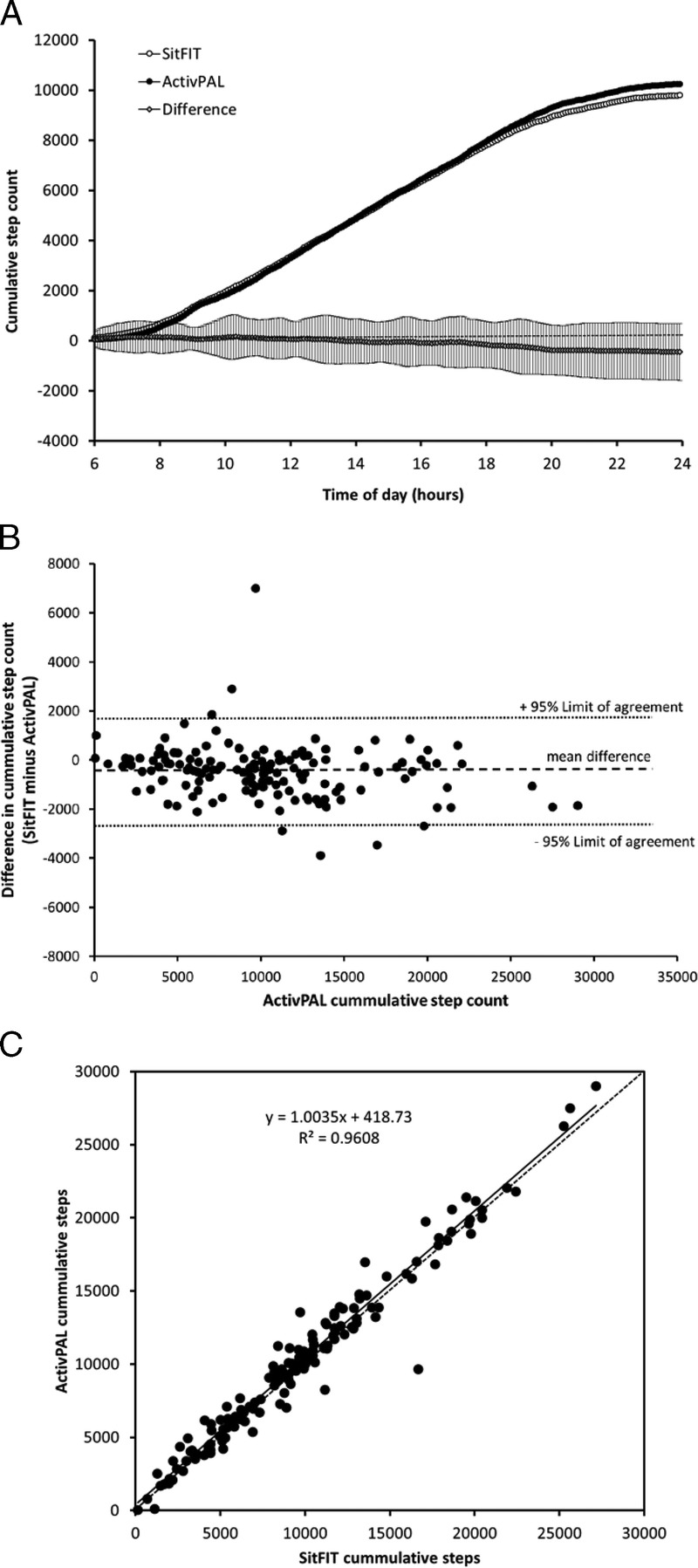
(Panel A) Cumulative step counts and differences in cumulative step counts over the course of the day measured using the SitFIT and activPAL devices. *N* = 145, values are mean for step counts for each device and mean ± SD for the difference in step count. (Panel B) Scatterplot showing the relationship between daily step counts measured using SitFIT and activPAL devices. *Solid line* is line of best fit; *dotted line* is line of equality; *N* = 145. (Panel C) Bland–Altman plot of difference in step counts between SitFIT and activPAL devices against ActivPAL (criterion standard) step counts. *N* = 145, *central dotted line* represents mean difference between devices; *outer dotted lines* represent 95% limits of agreement.

Over the 145 measurement days, mean (± SD) daily wear time for the SitFIT was 16.1 ± 4.2 h and for the ActivPAL was 22.9 ± 3.0 h. The median time for putting on the SitFIT in the morning was 07:35; the median time for removing it in the evening was 22:55. Comparisons between the SitFIT and ActivPAL for step-counts and sedentary time were made over the period when both devices were worn on each day. Figure 3A shows mean cumulative step count values over the 145 d measured using SitFIT and activPAL devices with the mean ± SD for differences in cumulative step counts between the two devices over the course of the day. Throughout the day, differences in cumulative step count between the devices were small, with no clear bias in either a positive or negative direction. Mean (± SD) daily step counts for the two devices over the 145 observation periods are shown in Table 1. Figure 3B shows a Bland–Altman plot of the mean difference and 95% limits of agreement for 24-h step counts between SitFIT and ActivPAL devices, with values summarized in Table 2. Overall, mean step counts were approximately 4% lower with the SitFIT than ActivPAL, with the 95% limits of agreement for step counts between the devices ranging from −2667 to +1817 steps per day. Mean absolute error in step count for the SitFIT compared with the ActivPAL was 826 steps per day. Step counts between the two devices differed by less than 1000 steps per day on 69% (100 of 145) of days and by less than 2000 steps on 94% (137/145) of days. Pearson correlation between step counts for the two methods was very high (*r* = 0.98, *r*^2^ = 0.96), with no obvious bias from the line of equality (equation of regression line, *y* = 1.0035*x* + 418.35) (Fig. 3C).

**TABLE 2:**
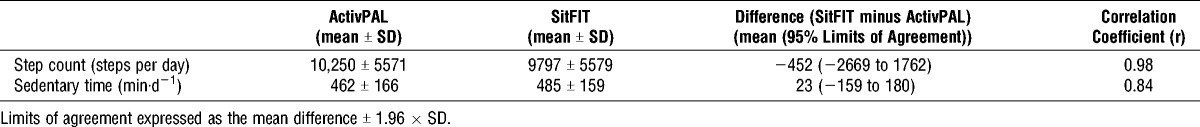
Comparison of ActivPAL and SitFIT derived measures of step counts and sedentary time over one hundred forty-five 24-h observation periods.

**FIGURE 4:**
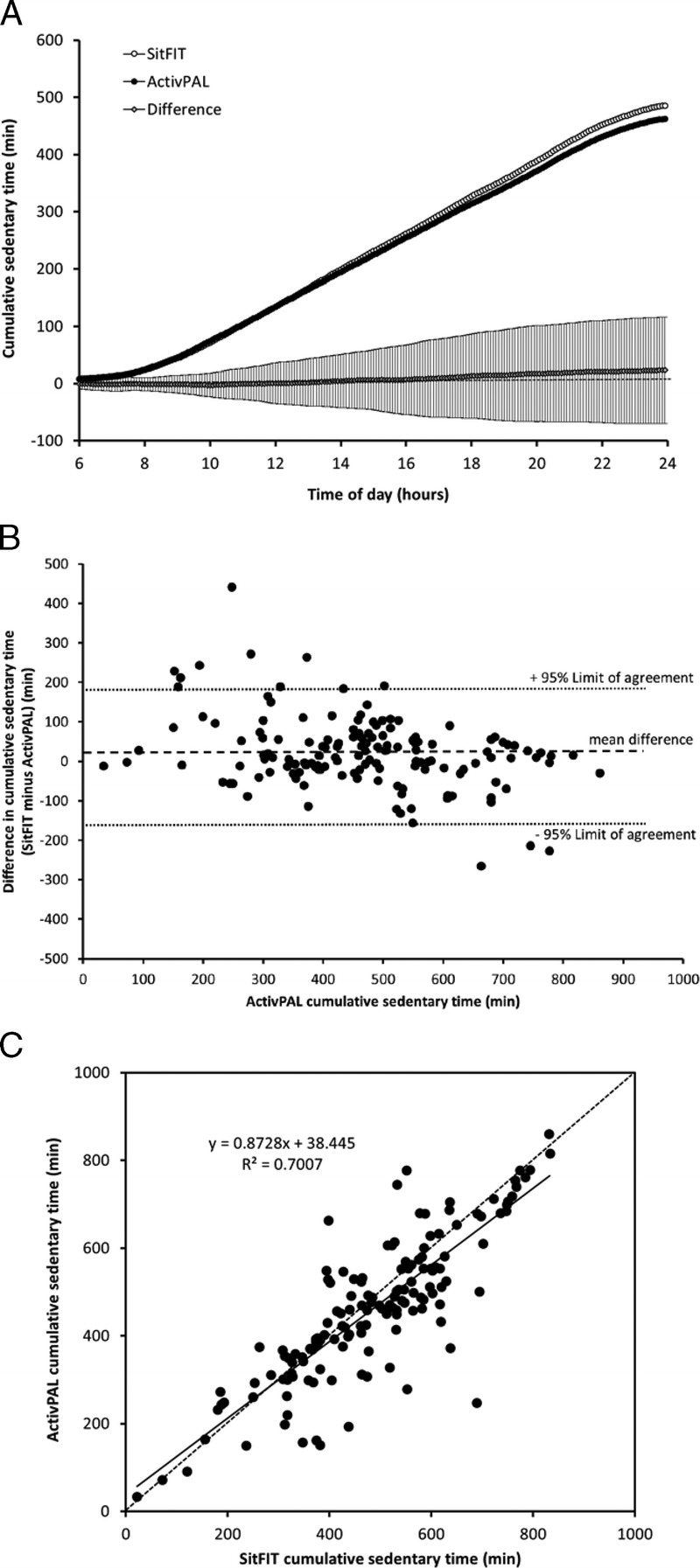
(Panel A) Cumulative sedentary time and differences in cumulative sedentary time over the course of the day measured using the SitFIT and activPAL devices. *N* = 145, values are mean for sedentary time for each device and mean ± SD for the difference in step count sedentary time. (Panel B) Scatterplot showing the relationship between daily sedentary time measured using SitFIT and activPAL devices. *Solid line* is line of best fit; *dotted line* is line of equality; *N* = 145. (Panel C) Bland–Altman plot of difference in sedentary time between SitFIT and activPAL devices against ActivPAL (criterion standard) sedentary time. *N* = 145, *central dotted line* represents mean difference between devices; *outer dotted lines* represent 95% limits of agreement.

Figure 4A shows mean cumulative sedentary time values over the 145 d measured using SitFIT and activPAL devices with the mean and standard deviation for differences in cumulative sedentary time between the two devices. Over the course of the day, there was no clear bias in sedentary time between the two devices: mean (±SD) daily values for sedentary time for the SitFIT and activPAL are shown in Table 2. A Bland–Altman plot of the mean difference and 95% limits of agreement for sedentary time are shown in Figure 4B, with values summarized in Table 2. Overall, mean sedentary time was approximately 5% higher with the SitFIT than activPAL, with 95% limits of agreement ranging from −159 min to +180 min·d^−1^. Mean absolute error in sedentary time for the SitFIT compared with the ActivPAL was 66 min·d^−1^. Sedentary time measures between the two devices differed by less than 60 min on 61% (89/145) and by less than 120 min on 86% (125/145) of days. Correlation between upright time for the two methods was high (*r* = 0.84, *r*^2^ = 0.70), although lower than observed for step count, with the equation of the regression line being close to the line of equality (*y* = 0.8728*x* + 38.445) (Fig. 4C).

## DISCUSSION

The aim of this article was to describe the development and validation of the SitFIT—a novel pocket-worn device to measure and provide real-time feedback on sedentary behavior and stepping activities. Although the SitFIT was initially designed for use in the EuroFIT trial (23), it can be used as a monitoring tool for sedentary behavior and stepping in widespread settings. Novel algorithms were developed to detect sitting and upright postures, which accounted for changes in device orientation within the pocket, and the accuracy of the SitFIT for measurement of step counts and sedentary behavior was assessed under free-living conditions. Our data revealed that the SitFIT had excellent validity for counting steps, with a mean difference in step counts between SitFIT and activPAL devices of approximately 4%, a correlation coefficient for step counts between the two devices of 0.98, and daily step counts differing between the two devices by less than 2000 steps on 94% of measurement days. Previous studies have reported that the most accurate commercially available pedometers have a 95% confidence interval for free-living 24-h step counts of approximately ±3000 to 4000 steps per day compared with a criterion measure and suggested that devices with mean differences in step counts within ±10% of the criterion measure have acceptable validity (27,28). More recently, correlation coefficients with criterion measures for 24-h steps counts for commercially available wearable activity monitors have been reported in the range of 0.94 to 0.99 with 95% confidence intervals for the difference in 24-h step counts typically within approximately ±1000 to 3000 steps per day (29). Thus, overall, these data indicate that the SitFIT device has excellent validity for measuring step counts under free-living conditions which is at least as good as other devices on the market.

Although there are a number of acceptable options available which monitor and provide feedback on indices of physical activity, such as step counts, devices which provide real-time feedback on sedentary behavior are more limited. The activPAL is generally regarded as the criterion standard device for the measurement of sedentary behavior (24,25): one version of this device—the activPAL VT (http://www.paltechnologies.com/products/)—provides vibrotactile feedback to the wearer when they have sat continuously for 15 or 30 min to provide information and a prompt to stand up. The SitFIT builds on activPAL VT in two important ways. First, its pocket location is more amenable to long-term wear than having a device affixed to the front of the thigh, and second, it has a display which provides real-time feedback on step count and time spent sitting (or upright)—analogous to a pedometer—which can thus be used to work toward daily targets. The LUMOback activity tracker (LUMO Bodytech, Mountain View, CA)—a device worn as a belt around the waist which is synced to a smartphone to provide feedback on sitting, standing, and stepping—was used in one randomized controlled trial as an intervention tool to facilitate reductions in sitting time amongst office workers (30). However, this device, which was originally developed as a posture monitor, has now been discontinued by the manufacturer, and its replacement, the Lumo Lift, with its placement near the collarbone is not suitable for objective monitoring of sitting behavior (http://www.lumobodytech.com/lumo-back/, accessed 14.03.17). Most other devices purporting to provide feedback on sedentary behavior to the user do so by equating sedentary time as a lack of dynamic movement, rather than by measurement of a sitting posture (21,22), and therefore do not provide a direct measurement of sedentary behavior in line with the Sedentary Behavior Research Network definition (1). This has potentially important implications, as these other devices would record a period of quiet standing as being sedentary, and there is increasing evidence that breaking up sitting with periods of quiet standing can produce metabolic benefits (13–15,31). Thus, such devices would not be able to provide effective feedback on a standing desk intervention, for example. Therefore, there is a clear need for a simple device that can provide users with feedback on sitting behavior, and the SitFIT addresses this gap.

The accuracy of the SitFIT for measurement of time spent sitting was also very good. Mean sedentary time as measured by the SitFIT and activPAL differed by approximately 5%, with a correlation coefficient between the two measures of 0.84. This compares favourably with validation of the LUMOback against the activPAL which reported a mean difference of 9.5% between the two devices for measurement of sedentary behavior over a 24-h cycle (22). The difference in daily sitting time between the SitFIT and activPAL was less than 60 min on 61% of day and less than 120 min on 86% of days. Other devices use an acceleration-based, rather than posture-based, approach to estimate time spent sedentary (21,22) and thus cannot distinguish between sitting and quiet standing. When such devices are validated against the activPAL, their accuracy in determining sedentary behavior is considerably poorer (22), which limits their potential for use in intervention aimed at reducing sitting time. It is of note that the accuracy of the SitFIT in measuring step counts was somewhat higher than its accuracy in determining time spent sitting. This is understandable given the greater technical challenges associated with quantification of sitting time compared with quantification of step count. The pocket location of the SitFIT has a number of advantages with respect to long-term usability: it can be carried inconspicuously, it is not directly attached to the skin (as the activPAL is) and is easily accessible for the provision of feedback to the user. However, as the SitFIT is free to move and change orientation in the pocket, the technical challenge of detecting posture allocation (sitting vs upright) is substantially greater than for the detection of steps; this contrasts with the criterion standard activPAL where the location and orientation of the device on the thigh is constant. To address this problem, an algorithm was developed to account for the random orientation of SitFIT in the pocket, as described in the methods. In this context, we feel that the validity of this algorithm, assessed here under real-world free-living conditions, for detection of sitting and upright time (the latter simply being wear time minus sitting time) is excellent and certainly acceptable for use as a tool to provide users with feedback on sedentary behavior in behavior change intervention programs.

This study provides an important first step in validating the SitFIT but further work is needed to validate the device in groups of users other than middle-age men and to provide construct as well as criterion validity for the device. There are also some limitations with the SitFIT which need to be considered. Firstly, as the device is pocket-worn, it may not be suitable for use for people who do not usually wear trousers with front pockets. To address this issue, a new device called the Activator, which is based on the same sensing platform as the SitFIT, but can be attached to clothing or worn discretely on the thigh using an integrated elastic loop (in addition to the option of being pocket-worn), is currently being developed by PAL Technologies. Second, although the accuracy of the SitFIT for measurement of sedentary behavior is acceptable for providing user feedback in the context of a behavior change intervention, it is not equivalent to the ActivPAL in this context, so for measurement of sedentary behavior as a research outcome, it should not be considered to be an ActivPAL replacement.

For the output display on the SitFIT, we deliberately chose to provide users with simple, actionable, feedback with the aim of facilitating behavior change. Pedometers, which provide a simple output of step count, are effective at increasing physical activity (17–19): with the SitFIT, we sought to provide an additional simple summary measure of sedentary time which could be used for goal setting and feedback. Further work is needed to validate the device for other outputs, such as number of sit-to-stand transitions, which have been shown to be associated with metabolic outcomes (15,32) and are a viable target for a sedentary behavior change intervention. In addition, further work is needed to develop and validate outputs related to intensity of physical activity, in addition to total step count, for the SitFIT. Increasing the number and complexity of data outputs would necessarily complicate the output display, and end-user input would be needed to develop the best ways of visualizing such data outputs for the user. Trials would also be needed to determine whether provision of more detailed feedback beyond step count and total sedentary time would lead to greater behavior change.

In conclusion, the SitFIT—a novel device to monitor and provide real-time feedback of stepping and sedentary behavior—has excellent validity for the measurement of step counts and sitting and upright time. Although there are a number of devices available which can provide feedback to the user on step counts, there is a lack of devices available which can provide feedback on time spent sitting and being upright. Thus, the SitFIT addresses a clear need for a device that can be used as a tool to provide feedback to the user on sedentary behavior to facilitate behavior change. As such, the SitFIT can be considered to be a complementary device to the ActivPAL, which remains the criterion standard device for measurement of sedentary behavior as a research outcome. Randomized controlled trials—such as the EuroFIT study (23)—are now needed to determine the effectiveness of such technology-supported approaches for eliciting long-term sedentary behavior change.
